# First Human Usutu Virus Reported in Asti (Piedmont, Italy, August 2022) and Early Follow-Up

**DOI:** 10.3390/tropicalmed7120443

**Published:** 2022-12-17

**Authors:** Tommaso Lupia, Fabio Paolo Marletto, Ilvana Tiziana Scuvera, Roberta Bosio, Barbara Rizzello, Valentina Fornari, Daniela Maria Luisa Vivenza, Valeria Ghisetti, Maria Teresa Brusa, Silvia Corcione, Francesco Giuseppe De Rosa

**Affiliations:** 1 Unit of Infectious Diseases, Cardinal Massaia, 14100 Asti, Italy; 2 Blood Transfusion Centre, Cardinal Massaia, 14100 Asti, Italy; 3 Department of Medical Sciences, Infectious Diseases, University of Turin, 10126 Turin, Italy; 4 Microbiology Unit, Amedeo di Savoia Hospital, 10100 Turin, Italy; 5 School of Medicine, Tufts University, Boston, MA 02111, USA

**Keywords:** Usutu, *Flaviviridae*, arbovirus, hepatitis, USUV

## Abstract

The Usutu virus (USUV) has recently attracted the attention of scientists because of its rapid spread across Europe and its growth over the previous seasons in Italy. Here, we describe the first case of USUV infection in Asti, Piedmont region, Italy. The patient remained asymptomatic in the acute phase and during the early follow-up, despite a mild increase in liver enzymes. The prompt diagnosis in this patient was due to positive qualitative PCR for WNV blood-donor screening with negative RT-PCR of WNV and positive USUV-RNA following the confirmation test. Blood-donor screening and transmission risk monitoring are pivotal in following the spread of this Flavivirus in non-endemic countries, due to the high percentage of asymptomatic carriers.

## 1. Introduction

Usutu virus (USUV) has recently attracted the attention of scientists because of its rapid spread across Europe [1]. USUV is an arbovirus in the family *Flaviviridae*, genus Flavivirus and Japanese encephalitis sero-complex [2]. Some of the most dangerous human arboviruses belong to the family *Flaviviridae*, including the West Nile virus (WNV), dengue virus, yellow fever virus, Zika virus, and Japanese encephalitis virus [2].

USUV is a small, spherical, enclosed virus with a positive-sense ribonucleic acid (RNA) genome of approximately 12 kilobase pairs in length [3]. Similar to other flaviviruses, viral replication takes place in the cytoplasm of infected cells [1,2].

Using phylogenetic analysis of the non-structural 5 (NS5) gene, researchers have classified USUV strains into eight distinct genetic subclades, including three African (Africa 1–3) and five European subclades (Europe 1–5) [4]. Although USUV is naturally maintained in a bird–mosquito–bird cycle, the identification of the virus or its antibodies has occasionally been reported in people, horses, and other mammals [5].

The name of USUV can be traced to the Usutu River in Eswatini (formerly Swaziland), Southern Africa [1]. In 1959, by inoculating new-born mice intracerebrally with samples derived from field-caught *Culex neavei* mosquitoes, McIntosh discovered USUV as part of a larger investigation into virus prevalence in South African arthropods [6].

After being discovered in South Africa, USUV has been found in several other nations across the continent, including the Central African Republic, Senegal, the Ivory Coast, Nigeria, Uganda, Burkina Faso, Tunisia, and Morocco [7].

Moreover, the first occurrence of USUV in Europe was linked to the mortality of Eurasian blackbirds in Italy in 1996, marking the virus’s first known appearance in Europe [8]. Various European nations, especially Germany, Austria, Belgium, Croatia, Spain, France, Greece, Hungary, Italy, the Czech Republic, Serbia, and Switzerland, recorded cases of USUV infection in mosquitoes, birds, and horses until 2015 [3].

Since 2008, surveillance programmes for arboviruses have been set up in Northern Italy, mainly for WNV control, but the screening of mosquitoes (*Culex pipiens*) and birds provides evidence of the co-circulation of different USUV lineages in the surveyed territory [9].

In fact, in addition to USUV’s rapid expansion in Western Europe in 2018, a major WNV pandemic occurred, resulting in 1503 human cases and 181 deaths across a dozen of European nations [10]. Moreover, data regarding the spread of autochthonous WNV in Asti, Piedmont (Italy), in 2018 were recently reported [11].

In humans living in Italy, evidence of both asymptomatic and symptomatic USUV infections has been reported in recent years [12,13]. Interestingly, prior to this report, we are unaware of any other USUV infections reported in Asti, Piedmont region, Italy.

Here, we describe the first case of USUV infection in a patient with a mild case of hepatitis in Asti, Piedmont region, Italy, in August 2022, and the first three months of follow-up.

## 2. Detailed Case Description

In August 2022, a 48-year-old Caucasian male arrived at our hospital’s blood transfusion centre in Northwest Italy for a routine exam before donating blood. His routine virological screening tests for serological human immunodeficiency virus, hepatitis B virus and hepatitis C virus, and Treponema pallidum spp. pallidum were negative. Moreover, due to the epidemiology in our region, a rapid WNV qualitative polymerase chain reaction (PCR) test was performed with a positive result. The positive sample was sent to the virological referral centre of our Piedmont region in the Amedeo di Savoia Hospital in Turin. The quantitative test for WNV resulted in a negative WNV-RNA count; however, a second quantitative test for USUV (in-house real-time PCR, RT-PCR) of the 3′ untranslated (UTR) region was performed, with a positive count of 637 cps/uL. The patient was called back for a clinical visit and interview. He worked as a decorator and lived in a country house nearby the city centre. He did not own any pets and denied contact with animals. His principal hobby was trekking in the mountains. He had never travelled outside of Italy, and his last journey to Naples, Italy, was in July, 33 days prior to this clinical visit. During the visit, the patient was apyretic and eupneic. He denied fever, rash, myalgias, arthralgias, nausea, vomiting, and neurological symptoms. He complained of experiencing one day of asthenia, but he linked this to a troublesome day at work. His past medical history was unremarkable despite hypercholesterolaemia, for which he began a low-lipid diet. Furthermore, a blood sample was taken to check for biochemical markers. His total blood count was normal (Table 1), although we found a mild increase in liver glutamic-pyruvic transaminase (GPT) and glutamic-oxaloacetic transaminase (GOT) levels with normal cholestasis markers (Table 1).

The patient was followed for three months as an outpatient, and no new signs or symptoms linked to his USUV infection appeared. Due to his mild hepatitis, an abdominal ultrasound was performed, but did not indicate any abnormalities. No lumbar puncture was proposed to the patient, due to his complete absence of neurological symptoms, and no serological tests or RT-PCR on other samples were processed, due to the lack of available test kits.

One month after the USUV RT-PCR positive result was recorded, a new blood sample was taken with a negative RNA count. Moreover, a complete blood count was performed 30 days after the initial visit, indicating a normal lymphocyte count and liver enzymes within normal limits.

In November 2022, three months after the USUV diagnosis, a phone interview was performed with the patient to critically review his medical condition over the last three months. The phone interview confirmed the completely asymptomatic course of the USUV infection. No new symptoms or signs occurred.

## 3. Discussion

In this case report, we describe the first USUV infection in Asti, Piedmont, Italy, which occurred in August 2022.

Several cases of USUV have been reported in Europe and Italy in the last ten years [3,4,13]. Interestingly, in the latest Italian epidemiological report on USUV and WNV, the former was identified in mosquitoes captured in up to eight Italian regions, including Piedmont, and in wild birds in Emilia Romagna, Veneto and Tuscany [14]. Moreover, in 2022, until November 2022, six cases of USUV were reported in Italy; these were considered mostly asymptomatic cases, despite the fact that complete clinical data were not available for these patients [14].

**Figure 1:**
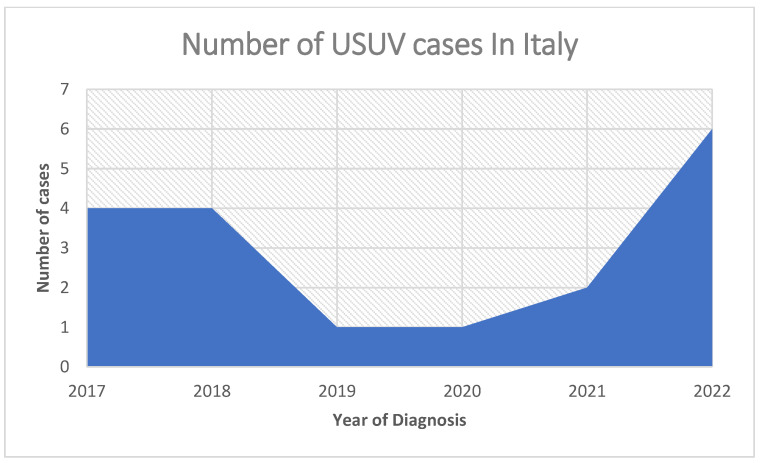
Number of Usutu virus infections in Italy between 2017 to 2022.

In Italy, between 2017 and 2022, according to epidemiological reports [15,16,17,18,19], cases of USUV have shown up annually, as reported in Figure 1, and appear to be growing.

An enzootic cycle that involves mosquitoes as vectors and wild birds as amplifying hosts keeps USUV alive in the environment [20]. This cycle is responsible for the maintenance of USUV. The endemic nature of USUV in northern Italy was brought to light by surveillance actions carried out in previous years [20]. According to the findings of Zecchin and his colleagues [21], who investigated the spatial spread of the virus in Europe, Italy was the primary contributor to the spread of USUV to the other European countries that are geographically adjacent to it.

Unfortunately, few cases of USUV infection in humans have been documented in the literature; hence, little is known about the spectrum of clinical manifestations. Despite this, the clinical presentation of autochthonous USUV infection in Europe is very heterogeneous, but most patients are completely asymptomatic [20]. Symptoms vary from mild to moderate (e.g., fever, rash and headache) to severe signs of infection (e.g., meningism and neuropathy), especially in immune-depressed patients; these neuroinvasive complications have also been reported in Italy [20].

Based on the uncertainty in the clinical course and presentation, we decided to follow up the patient with laboratory monitoring and clinical consultations during the first three months after the infection (Table 1).

Laboratory tests on our patient revealed a mild increase in GPT and GOT. In previously reported USUV cases, liver involvement ranged from asymptomatic liver inflammation to fulminant hepatitis [21,22]. In our report, the course of infection was completely asymptomatic from the beginning, despite mild asthenia that could theoretically be linked to mild liver inflammation. Moreover, by following the patient for three months after the infection, we confirmed the benign course of the patient’s USUV infection, without any new symptoms or signs reported during the phone interview three months after the infection.

Interestingly, Kuchinsky and colleagues [23] have demonstrated that the virulence and genetics of strains of USUV are distinct from one another, and that there are significant genetic differences across the strains that are circulating in Europe. The authors studied liver histopathological findings in cohorts of mice. In the liver tissues of animals that had been infected with strains of the Usutu virus from Uganda, Spain, Senegal, or South Africa, prominent cell death as well as varied inflammation were reported. In the liver tissues of the animals that had been infected with the strain from the Netherlands, mild inflammation was reported [23]. Mice that were infected with any of the four African strains studied or the single European strain that originated in Spain all succumbed to the infection with identical survival rates and histology in their tissues. Surprisingly, a European strain that originated in the Netherlands caused just 12% mortality and considerably less histopathology in the tissues of mice, when compared to animals who had been injected with one of the other strains. Mice implanted with the recent African strains had the highest levels of viral replication, whereas mice inoculated with the strain from the Netherlands had the lowest levels of viral replication [23]. These findings suggest the potentially different virulence and clinical presentation of USUV infections in Europe in relation to the circulating strain and also differences between autochthonous and imported strains.

Infection with USUV can be identified through the detection of particular antibodies, the viral RNA genome, and/or through the isolation of the virus in cell culture. There are currently no validated commercial serological or molecular assays available for purchase.

The prompt diagnosis in this patient was due to positive qualitative PCR for WNV blood-donor screening. Cross-reactions between WNV and USUV serology and qualitative PCR tests and misdiagnosis of USUV infection are not uncommon, as reported by Percivalle et al. In our case, a second-line in-house quantitative RT-PCR reflex test was pivotal to reach the diagnosis [24].

A USUV diagnosis at this time is associated with a better prognosis than WNV infections, probably due to the lower neuroinvasive risk, but from an epidemiological point of view, reports of infections are of high importance, especially in regions that, until now, have reported a low case rate [25].

The under-recognition of USUV infections in humans may have been caused, in part, by the restricted availability of diagnostic testing, the strong serological cross-reactivity, and the co-circulation with WNV. Human infections are regarded as sporadic. However, over the past few years, there has been a rising number of reports regarding USUV human infections. These reports have been notably prevalent in nations that have active monitoring plans for WNV, which has led researchers to postulate that USUV may play a role as a human pathogen.

## 4. Conclusions

In conclusion, USUV infections are growing in Europe, despite the current low incidence rates in Italy. Blood-donor screening and risk of transmission are pivotal at this moment to follow the spread of this Flavivirus. USUV cases, despite the low risk of symptomatic and severe cases, should be followed up in the first few weeks after diagnosis.

## Figures and Tables

**Table 1:** Laboratory and virological features at diagnosis and during follow-up.

	August (at Diagnosis)	September (1-Month Follow-Up)	November (3-Month Follow-Up)
WBC count (U/microL)	5	5.4	NA
Lymphocites (U/microL)	1.53	1.43	NA
Monocytes (U/microL)	0.29	0.35	NA
Neutropenia (U/microL)	3.1	3.55	NA
RBC (U/microL)	5.07	4.72	NA
Hb (g/dL)	15.5	14.5	NA
PLTS (U/microL)	187	171	NA
Creatinine (mg/dL)	0.91	NA	NA
GOT	39	27	25
GPT	56	35	35
C-RP (mg/L)	0.06	0.05	NA
WNV-RNA (qualitative)	Positive	Negative	Negative
WNV-RNA (quantitative)	Negative	Negative	Negative
USUV-RNA (cps/mL)	637	Negative	Negative

Abbreviations: WBC: white blood cell; U: unit; RBC: red blood cell; Hb: haemoglobin; PLTS: platelets; GPT: glutamic-pyruvic transaminase; GOT: glutamic-oxaloacetic transaminase; C-RP: C-reactive protein; WNV: West Nile virus; USUV: Usutu virus; RNA: ribonucleic acid; cps: copies; NA: not available.

## Data Availability

Not applicable.

## References

[1] Clé M., Beck C., Salinas S., Lecollinet S., Gutierrez S., Van de Perre P., Baldet T., Foulongne V., Simonin Y. (2019). Usutu virus: A new threat?. Epidemiol. Infect..

[2] Gould E., Solomon T. (2008). Pathogenic flaviviruses. Lancet.

[3] Vilibic-Cavlek T., Petrovic T., Savic V., Barbic L., Tabain I., Stevanovic V., Klobucar A., Mrzljak A., Ilic M., Bogdanic M. (2020). Epidemiology of Usutu Virus: The European Scenario. Pathogens.

[4] Roesch F., Fajardo A., Moratorio G., Vignuzzi M. (2019). Usutu Virus: An Arbovirus on the Rise. Viruses.

[5] Khare B., Kuhn R.J. (2022). The Japanese Encephalitis Antigenic Complex Viruses: From Structure to Immunity. Viruses.

[6] McIntosh B.M. (1985). Usutu (SAAr 1776); nouvel arbovirus du groupe B. Int. Cat Arboviruses.

[7] Nikolay B., Diallo M., Boye CS B., Sall A.A. (2011). Usutu virus in Africa. Vector-Borne Zoonotic Dis..

[8] Weissenböck H., Bakonyi T., Rossi G., Mani P., Nowotny N. (2013). Usutu Virus, Italy, 1996. Emerg. Infect. Dis..

[9] Calzolari M., Chiapponi C., Bonilauri P., Lelli D., Baioni L., Barbieri I., Lavazza A., Pongolini S., Dottori M., Martin A.M.M. (2017). Co-Circulation of Two Usutu Virus Strains in Northern Italy between 2009 and 2014. Infect. Genet. Evol..

[10] European Centre for Disease Prevention and Control (ECDC) (2018). West Nile fever in Europe in 2018. https://ecdc.europa.eu/en/publications-data/west-nile-fever-europe-2018-human-cases-compared-previousseasonsupdated-23.

[11] Lupia T., Libanore V., Corcione S., Fornari V., Rizzello B., Bosio R., Stroffolini G., Bigliano P., Fontana S., Patti F. (2022). Autochthonous West Nile Virus Infection Outbreak in Humans (Asti, Piedmont, Italy, August-October 2018) and Long-Term Sequelae Follow-Up. Trop. Med. Infect. Dis..

[12] Pacenti M., Sinigaglia A., Martello T., De Rui M.E., Franchin E., Pagni S., Peta E., Riccetti S., Milani A., Montarsi F. (2019). Clinical and Virological Findings in Patients with Usutu Virus Infection, Northern Italy, 2018. Eurosurveillance.

[13] Zecchin B., Fusaro A., Milani A., Schivo A., Ravagnan S., Ormelli S., Mavian C., Michelutti A., Toniolo F., Barzon L. (2021). The central role of Italy in the spatial spread of USUTU virus in Europe. Virus Evol..

[14] https://www.epicentro.iss.it/westnile/bollettino/Bollettino_WND_2022_20.pdf.

[15] https://www.epicentro.iss.it/westnile/bollettino/Bollettino_WND_2020_16.pdf.

[16] https://www.epicentro.iss.it/westnile/bollettino/Bollettino_WND_2021_19.pdf.

[17] https://www.epicentro.iss.it/westnile/bollettino/Bollettino-WND-N16-25nov2019.pdf.

[18] https://www.epicentro.iss.it/westnile/bollettino/Bollettino%20WND_%20N.%2018%20%2015.%2011%202018.pdf.

[19] https://www.epicentro.iss.it/westnile/bollettino/Bollettino%20WND_08.11.2017.pdf.

[20] Gaibani P., Rossini G. (2017). An overview of Usutu virus. Microbes Infect..

[21] Zecchin B., Fusaro A., Milani A., Schivo A., Ravagnan S., Ormelli S., Mavian C., Michelutti A., Toniolo F., Barzon L. (2022). Italy as a Hotspot of Usutu Virus in Europe. Int. J. Infect. Dis..

[22] Gill C.M., Kapadia R.K., Beckham J.D., Piquet A.L., Tyler K.L., Pastula D.M. (2020). Usutu virus disease: A potential problem for North America?. J. Neurovirol..

[23] Kuchinsky S.C., Hawks S.A., Mossel E.C., Coutermarsh-Ott S., Duggal N.K. (2020). Differential pathogenesis of Usutu virus isolates in mice. PLoS Negl. Trop. Dis..

[24] Cavrini F., Gaibani P., Longo G., Pierro A.M., Rossini G., Bonilauri P., Gerunda G.E., Di Benedetto F., Pasetto A., Girardis M. (2009). Usutu virus infection in a patient who underwent orthotropic liver transplantation, Italy, August–September 2009. Eurosurveillance.

[25] Percivalle E., Cassaniti I., Sarasini A., Rovida F., Adzasehoun K.M.G., Colombini I., Isernia P., Cuppari I., Baldanti F. (2020). West Nile or Usutu Virus? A Three-Year Follow-Up of Humoral and Cellular Response in a Group of Asymptomatic Blood Donors. Viruses.

